# RCN1 deficiency inhibits oral squamous cell carcinoma progression and THP-1 macrophage M2 polarization

**DOI:** 10.1038/s41598-023-48801-2

**Published:** 2023-12-06

**Authors:** Han Liu, Haiyang Guo, Yuehan Wu, Qiannan Hu, Guangbing Hu, Huan He, Yaolin Yin, Xiaoxu Nan, Gaoren Lin, Jinpeng Han, Runzhe Zhao, Ying Liu

**Affiliations:** 1 Department of Stomatology, Affiliated Hospital of North Sichuan Medical College, Nanchong, China; 2 Department of Stomatology, North Sichuan Medical College, Nanchong, China; 3 Digestive Endoscopy Center, Affiliated Hospital of North Sichuan Medical College, Nanchong, Sichuan China; 4 Institute of Hepato-Biliary-Pancreatic-Intestinal Disease, North Sichuan Medical College, Nanchong, China

**Keywords:** Head and neck cancer, Oral cancer

## Abstract

Reticulocalbin 1 (RCN1), a calcium-binding protein located in the endoplasmic reticulum (ER) lumen, contains six conserved regions. Its main functions include maintaining intracellular homeostasis and regulating cell proliferation and apoptosis, and it plays an important role in the development of various tumours. However, the exact function of RCN1 in oral squamous cell carcinoma (OSCC) is not fully understood. Therefore, the aim of this study was to investigate the effects of RCN1 on the biological behaviour of OSCC and the regulation of tumour-associated macrophage (TAM) polarization. The expression of RCN1 in OSCC and normal oral mucosa was evaluated through bioinformatics analysis and immunohistochemical staining. The growth, migration, and invasion of OSCC cells were observed after knockdown of RCN1 using CCK-8 and Transwell assays. Apoptosis was detected by flow cytometry. The effect of tumour cell-derived RCN1 on the polarization of THP-1 macrophages was investigated by establishing a coculture model of THP-1 macrophages and OSCC cells. Additionally, changes in the expression levels of relevant proteins were detected using Western blotting. The upregulation of RCN1 in tumour tissues compared to normal oral mucosal tissues is associated with a poor prognosis and can be utilized as a prognostic indicator for OSCC. Knockdown of RCN1 inhibited the proliferation, migration, and invasion of OSCC cells. Additionally, knockdown of RCN1 in Cal-27 and SCC-25 cells resulted in inhibition of the M2 polarization of THP-1 macrophages. RCN1 knockdown inhibits OSCC progression and M2 macrophage polarization. Targeting RCN1 may be a promising approach for OSCC treatment.

## Introduction

OSCC is the most common malignant tumour of the head and neck; it originates from various parts of the oral cavity, such as the tongue, oral cavity, and pharynx. It constitutes approximately 90% of malignant epithelial tumours in the oral and maxillofacial region and is considered one of the most prevalent cancers worldwide^1,2^. Globally, in 2020 alone, there was a staggering 377,713 new cases and a total of 177,757 related deaths^3^. The primary treatment options for OSCC include surgery, chemotherapy, radiation therapy, or a combination of these approaches. However, the survival rate and quality of life for patients with advanced OSCC in the postoperative period have not significantly improved. This lack of progress can be attributed to postoperative recurrence and surgical trauma leading to oral and maxillofacial dysfunction^4,5^. Therefore, early detection, diagnosis, and treatment are crucial in improving the therapeutic outcomes for OSCC patients. Consequently, understanding the molecular mechanisms underlying OSCC development and progression has become a main research focus.

Reticulocyte calreticulin (RCN), also known as RCN1, is an endoplasmic reticulum inner membrane protein that was initially identified by Ozawa and colleagues in a mouse aberrant cell line in 1993^6^. RCN1 exhibits a high degree of homology (95%) between mice and humans, suggesting its conserved nature and potential significance in regulating normal cellular behaviour and function^7^. The expression of RCN1 has been increasingly observed in various tumour types, including hepatocellular carcinoma, prostate cancer, and highly invasive breast cancer^8,9^. In lung cancer, high levels of RCN1 expression have been linked to poor prognosis^10^. Additionally, in nasopharyngeal carcinoma, knockdown of RCN1 has been shown to enhance drug sensitivity in cancer cells^11^. These findings indicate that RCN1 may play a crucial role in tumour development and progression. However, studies investigating the role of RCN1 in OSCC are currently lacking. Therefore, our study aims to investigate the function of RCN1 in OSCC progression and assess its potential value as a molecular marker for OSCC.

Monocytes, originating in the bone marrow, migrate from the bloodstream to tumour where they differentiate into tumour-associated macrophages(TAMs)^12^. Under physiological conditions, TAMS can be categorized into two main states of differentiation: classically activated type 1 (M1) macrophages and alternatively activated type 2 (M2) macrophages. M1 TAMs primarily exert host defence functions and possess a proinflammatory phenotype characterized by the secretion of cytokines such as interleukin-12 (IL-12), IL-1, IL-23, and tumour necrosis factor alpha (TNFα). They contribute to antitumour functions^13^. In contrast, the M2 TAMs population promotes tissue regeneration and exhibits immunosuppressive properties by secreting cytokines such as IL-1β, IL-10, matrix metalloproteinase (MMP), transforming growth factor β (TGF-β), and vascular endothelial growth factor (VEGF). These factors contribute to immunomodulation and tissue remodelling^14,15^. Consequently, M2-like macrophages are potentially important mediators in the bidirectional communication between cancer cells and their microenvironment. Various studies have explored the specific mechanisms of communication between cancer cells and M2 TAMs^16^. However, the precise mechanisms through which tumour cells promote M2 TAMs polarization in the context of OSCC remain unknown.

In this study, our main finding were that RCN1 is overexpressed in OSCC and that this phenotype is associated with a poor prognosis for OSCC patients. Moreover, we investigated the functional role of RCN1 in OSCC progression and explored the underlying mechanisms. Our results demonstrated that RCN1 plays a significant role in promoting OSCC cell proliferation, migration, invasion, and M2 TAMs polarization. By elucidating that RCN1 influences OSCC progression, our study provides new insights into potential therapeutic strategies for the treatment of OSCC.

## Materials and methods

### Bioinformatics analysis

TIMER is a valuable resource (TIMER2.0 (cistrome.org)) that facilitates systematic analysis of immune infiltration in various cancer types^17–19^. In our study, we utilized this resource to examine the expression pattern of RCN1 in multiple tumours. UALCAN (UALCAN (uab.edu)), a comprehensive online analytical tool, for in-depth investigation of TCGA gene expression and survival analysis across 33 different cancer types^20,21^. We employed UALCAN to analyse the expression of RCN1 in HNSCC and assess its correlation with prognosis.

### Patient specimens

A total of 23 specimens of OSCC and 13 specimens of normal oral mucosal tissues were collected from patients who underwent their initial radical surgery for OSCC at the Department of Maxillofacial Surgery, Affiliated Hospital of North Sichuan Medical College, between September 2020 and December 2022 and had available clinicopathological information. None of these patients received preoperative chemotherapy or radiotherapy. All patients provided consent to participate, and ethical approval was obtained from the relevant ethics committees. The methods were performed in accordance with relevant guidelines and regulations.

### Immunohistochemical staining (IHC)

The tissues were initially fixed in formalin and subsequently embedded in paraffin. Following embedding, the tissues were cut into sections measuring 4 μm in thickness. These sections were then subjected to immunostaining using a diluted rabbit anti-human RCN1 monoclonal antibody (1:800, ab210404, Abcam). The immunostaining process took place at a temperature of 4 °C. RCN1 staining scores was assigned based on the percentage of stained area as follows: < 10% was denoted as 1, 10–50% was denoted as 2, 51–80% was denoted as 3, and > 80% was denoted as 4. The intensity of RCN1 staining was also graded as 1 (weak), 2 (moderate), or 3 (strong). The final RCN1 staining score was calculated by multiplying the staining area score by the staining intensity score, and the average value obtained was considered the IRS score.

### Cell culture

The human OSCC cell lines Cal-27 and SCC-25, as well as the human monocytic cell line THP-1, were obtained from the ATCC cell bank. Cal-27 cells were cultured in DMEM (VivaCell, Shanghai, China), SCC-25 cells were cultured in F12 medium (VivaCell), and THP-1 cells were cultured in 1640 medium (VivaCell). All media were supplemented with 10% FBS (foetal bovine serum), while 1% penicillin and 1% streptomycin antibiotics were added to the respective media. The cells were maintained in a cell culture incubator at 37 °C with a 5% CO_2_ environment.

### Cell transfection

To downregulate the expression of RCN1 in SCC-25 and Cal-27 cells, three lentiviral vectors specifically targeting the human RCN1 gene (shRCN1) and a negative control vector (vec) were designed and synthesized by Jikai Genetics (Shanghai, China). Approximately 2 × 10^5^ cells/well were seeded in six-well plates. After allowing the cells to attach to the culture plates, transfection was performed using HiTransG transfection reagent (Gikai Genetics, Shanghai, China). Subsequently, puromycin, an antibiotic, was employed to select for stable cell lines that underwent gene knockdown.

### RT‒qPCR

Total RNA was extracted from the cells using an RNA extraction kit from TIANGEN (Beijing, China). The extracted RNA was then reverse transcribed into cDNA using HiScript II Q RT SuperMix from Vazyme (Beijing, China). Subsequently, quantitative real-time polymerase chain reaction (qPCR) analysis was conducted using ChamQ™ SYBR Color qPCR Master Mix (Vazyme). The specific sequences of primers used for the qPCR analysis can be found in Table 1. The qPCR results were analysed using the 2^−ΔΔCt^ method.

**Table 1:** Primers used for qRT‒PCR.

Gene	Forward primer (5ʹ − 3ʹ)	Reverse primer (5ʹ − 3ʹ)
*CD206*	TCCGGGTGCTGTTCTCCTA	CCAGTCTGTTTTTGATGGCACT
*CD163*	TTTGTCAACTTGAGTCCCTTCAC	TCCCGCTACACTTGTTTTCAC
*CD68*	GGAAATGCCACGGTTCATCCA	TGGGGTTCAGTACAGAGATGC
*IL10*	GACTTTAAGGGTTACCTGGGTTG	TCACATGCGCCTTGATGTCTG
*ARG1*	GTGGAAACTTGCATGGACAAC	AATCCTGGCACATCGGGAATC
*RCN1*	AAACGGGTGCAGAAAAGATACA	AGGTAGTAACCATAGGTGGCTT
*GAPDH*	GGAGTCCACTGGCGTCTTCA	GTCATGAGTCCTTCCACGATACC

### Western blotting

Cells were lysed on ice using RIPA buffer (Solarbio, Beijing, China), followed by centrifugation and collection of the supernatant. The protein concentration was determined using a BCA kit (Solarbio). Subsequently, the proteins were denatured at 100 °C. Equal quantities of protein were separated by 10% sodium dodecyl sulfate–polyacrylamide gel electrophoresis (SDS-PAGE) and transferred onto polyvinylidene difluoride membranes. Membranes were subsequently blocked with 5% milk in TBS-T for 2 h at room temperature. The membranes were incubated overnight with the primary antibodies listed in Table 2. Afterwards, the membranes were incubated with secondary antibodies at room temperature. The blots were developed using an ECL kit, and densitometric analysis of the blots was performed using ImageJ.

**Table 2:** Antibodies for WB.

Target	Manufacturer	Catalogue number	Dilution factor
GAPDH	HuaBio	ET1601-4	1:80,000
RCN1	Abcam	ab210404	1:2000
Caspase 3	Abcam	ab184787	1:2000
Bcl-2	Abcam	ab196495	1:2000
E-cadherin	FineTest	FNab09408	1:1000
N-cadherin	FineTest	FNab05570	1:1000
Vimentin	FineTest	FNab02617	1:1000
MMP9	HuaBio	ET1704-69	1:500
AKT	HuaBio	ET1609-47	1:500
P-AKT	HuaBio	ET1607-73	1:500
CD206	HuaBio	ET1702-04	1:2000
IL 10	HuaBio	ER1911-19	1:1000
ARG1	HuaBio	ET1605-8	1:2000
HRP-labelled goat anti-rabbit IgG(H + L)	FineTest	FNSA-0004	1:5000

### Cell counting kit-8 (CCK-8) assay

The transfected cells were seeded into 96-well plates at a density of 1200 cells per well in a volume of 100 µL of medium. After incubation for 0, 24, 48, and 72 h, 10 µL of CCK-8 reagent (Vazyme) was added to each well. The plates were then incubated in the dark at 37 °C for 1 h. Following the incubation period, the absorbance value at 450 nm for each well was measured using an enzyme labelling instrument (Bio-Rad). The experiment was repeated three times to ensure reliable results.

### Transwell assays

For the migration assay, cells were seeded into the upper chamber of a transwell plate containing 200 µl of serum-free medium. For the invasion assay, Matrigel was added to the upper chamber before cell seeding. In both cases, complete medium (500 µl) was added to the lower chamber of the transwell plate. The plate was then incubated for 36 h to allow cell migration and invasion. After the incubation period, the cells on the bottom of the upper chamber were fixed with 4% paraformaldehyde for 30 min. Subsequently, they were stained with 0.1% crystal violet for another 30 min. Microscopy images of the cells were captured, and these images were further analysed using ImageJ software.

### Flow cytometry

The cells were seeded into six-well plates and incubated for 48 h. The cells were digested using EDTA-free trypsin. An apoptosis kit (KeyGEN BioTECH) was used for staining. The stained cells were subsequently analysed using flow cytometry (SONY).

### Macrophage polarization and coculture system

THP-1 cells were stimulated with phorbol 12-myristate 13-acetate (PMA, Sigma) for 48 h. When the cells differentiated, they were referred to as M0 macrophages. To establish a coculture system, THP-1-derived M0 macrophages were incubated with supernatants from Cal-27/SCC-25 cells that were transfected with sh-vec and shRCN1. This incubation took place over a period of 48 h. After incubation, the macrophages that were treated with the supernatants were collected for subsequent experiments.

### Statistical analysis

All data were analysed using GraphPad Prism version 9.0 statistical software. Each experiment was replicated three times. All data are presented as the mean ± standard deviation (SD). The differences between two or more groups were determined by Student's t test or one-way analysis of variance. p values < 0.05 were considered to have statistical significance.

### Ethics approval and consent to participate

All protocols in this study were approved by the Ethics Committee of the Affiliated Hospital of North Sichuan Medical College (ID:2022ER462-1), and informed consent was obtained from each patient.

## Results

### RCN1 is highly expressed in OSCC

**Figure 1:**
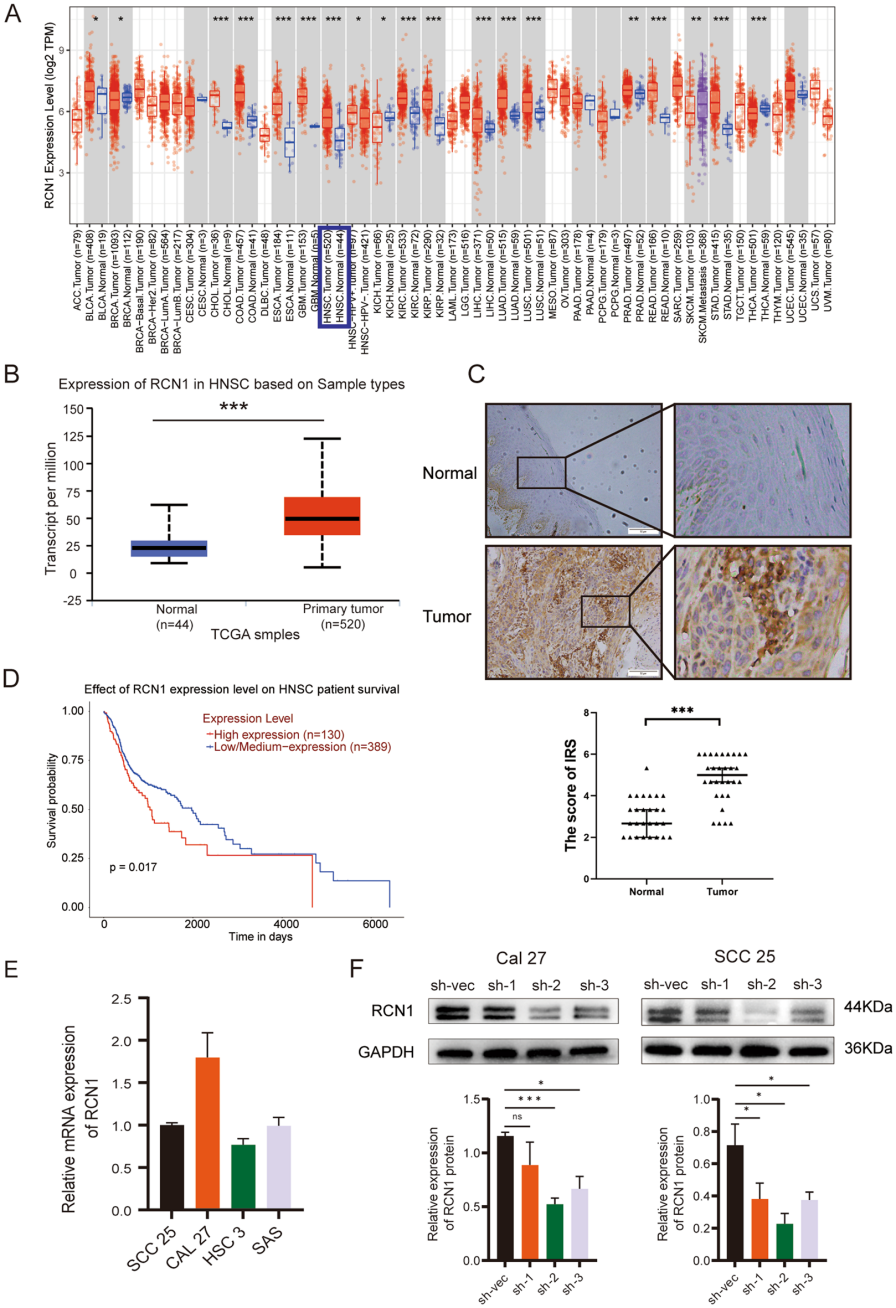
Relative expression and knockdown efficiency of RCN1 in OSCC cells. (**A**) TIMER database to analyse the expression of RCN1 in various tumours. (**B**) UALCAN database to analyse the expression of RCN1 in HNSCC. (**C**) Representative plots and statistical results of immunohistochemical analysis of RCN1 expression in OSCC tumour tissues as well as normal oral epithelial tissues. (**D**) UALCAN database analysis of the relationship between RCN1 and the prognosis of HNSCC patients. (**E**) RT‒PCR analysis of mRNA expression levels of RCN1 in different OSCC cell lines. (**F**) Western blot to verify the knockdown efficiency of RCN1. *p < 0.05, **p < 0.01, ***p < 0.001.

To unravel the expression pattern of RCN1 in various cancers, we obtained pan-cancer expression data from the TIMER database. Upon analysis, we observed that RCN1 exhibited high expression in HNSCC (Fig. 1A). To further investigate the expression of RCN1 in HNSCC, we utilized UALCAN data. Our analysis indicated that RCN1 expression was significantly higher in HNSCC tissues than in normal tissues (Fig. 1B). We also performed immunohistochemical staining on our collected OSCC samples and normal oral mucosal tissues, which confirmed the elevated expression of RCN1 in OSCC tissues compared to normal tissues (Fig. 1C). Moreover, to determine the association between RCN1 expression and patient prognosis, we conducted subsequent analysis using UALCAN data. Our findings revealed that patients with lower RCN1 expression had a significantly better prognosis than those with high RCN1 expression (Fig. 1D). In summary, the results from our study strongly indicate that RCN1 is highly expressed in OSCC patients and is associated with a poor patient prognosis.

To delve deeper into the contribution of RCN1 to the biological behaviour of OSCC cells, we evaluated the relative expression level of RCN1 mRNA in four OSCC cell lines (Fig. 1E). We chose Cal-27 and SCC-25 cell lines with relatively high RCN1 expression to establish the knockdown model of RCN1. Notably, according to Fig. 1F, the sh-2 sequence had the highest knockdown efficiency after transfection. Therefore, the sh-2 sequence was selected for subsequent experiments.

### RCN1 knockdown inhibits cell proliferation and promotes apoptosis

**Figure 2:**
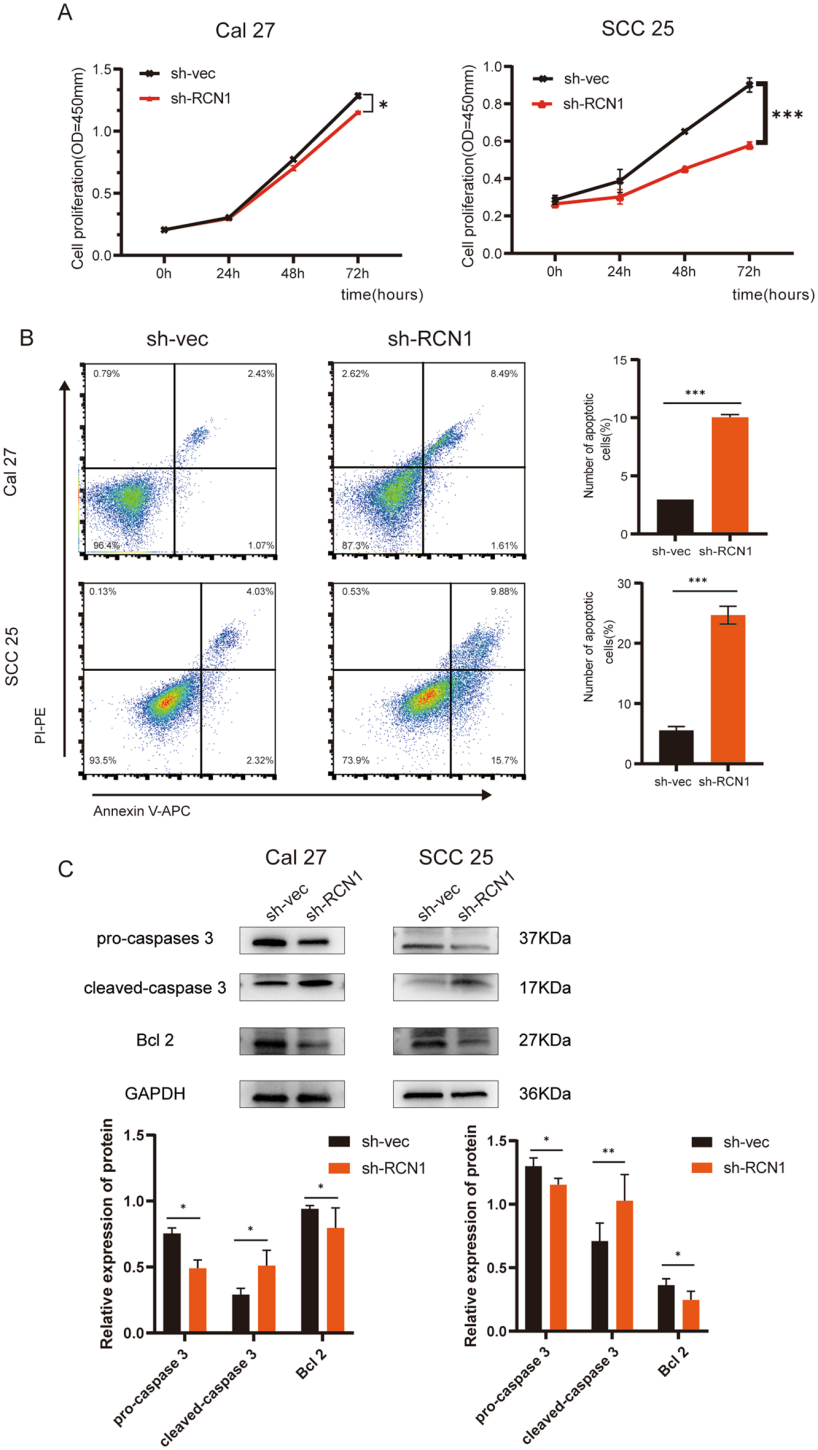
Knockdown of RCN1 inhibits OSCC cell proliferation and promotes apoptosis. (**A**) Results of CCK-8 experiments showing changes in the proliferation profiles of Cal-27 and SCC-25 cells after silencing RCN1. (**B**) Flow cytometry analysis of apoptotic changes in Cal-27 and SCC-25 cells after different treatments. (**C**) Detection of apoptosis-related proteins by immunoblotting assay. *p < 0.05, **p < 0.01, ***p < 0.001.

The effect of RCN1 on cell proliferation was investigated using a CCK-8 assay, which revealed a significant reduction in the proliferation ability of both OSCC cell lines after RCN1 knockdown (Fig. 2A). Flow cytometry analysis further demonstrated a substantial increase in apoptosis levels in both OSCC cell lines following RCN1 knockdown (Fig. 2B). These findings strongly indicate that the high expression of RCN1 in OSCC promotes cell proliferation while inhibiting apoptosis. Additionally, we examined the expression of Bcl-2, an apoptosis-inhibitory protein located in the mitochondria, and caspase 3, whose cleavage is the most important and final step in the process of apoptosis (Fig. 2C). The results showed that RCN1 knockdown led to a significant decrease in Bcl-2 expression, a notable decrease in pro-caspase 3 expression, and a considerable increase in cleaved caspase 3 expression. These results further support the notion that RCN1 promotes OSCC cell proliferation by inhibiting apoptosis.

### RCN1 knockdown inhibits cell migration and invasion

**Figure 3:**
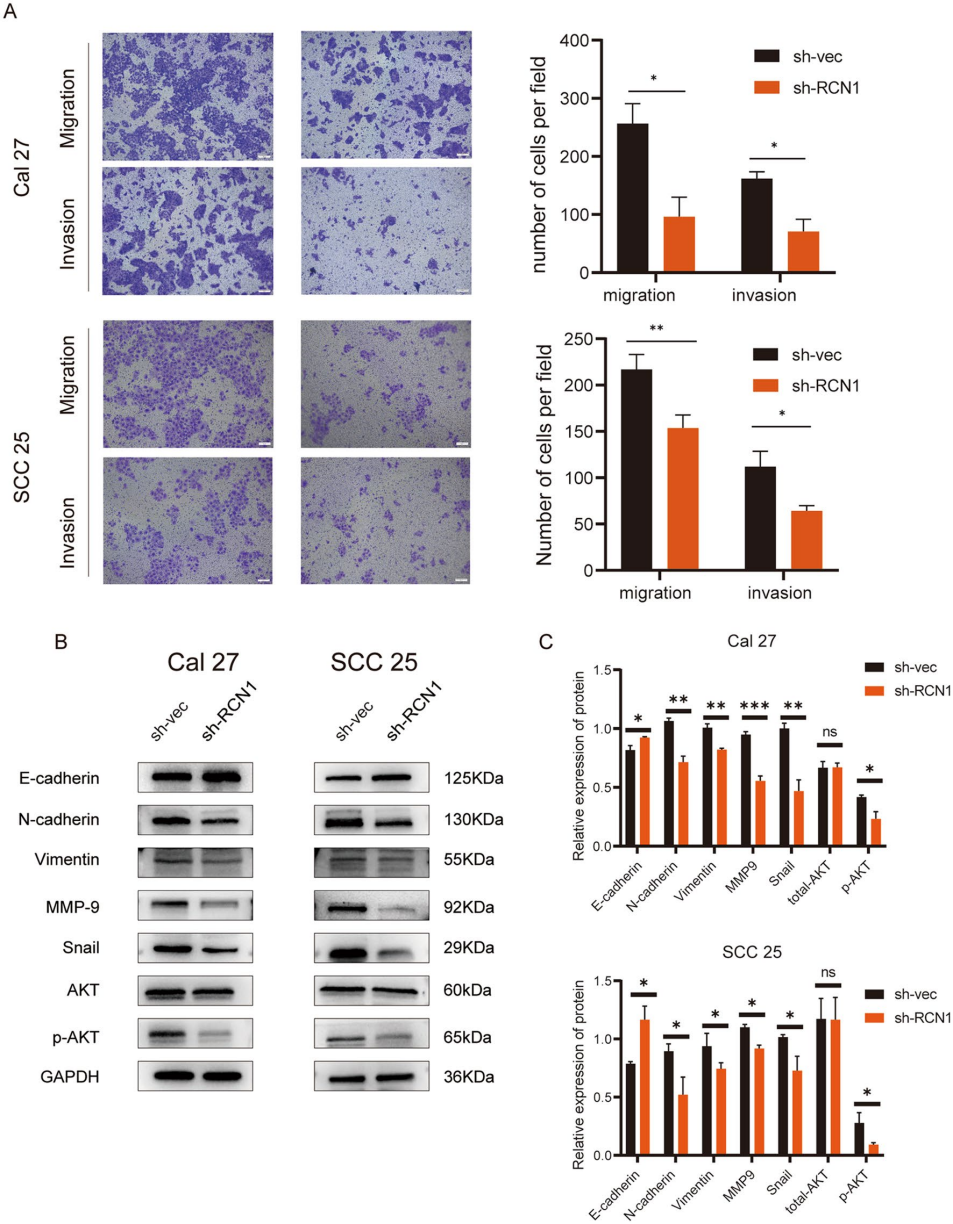
Knockdown of RCN1 inhibits the migration and invasion of OSCC cells by regulating EMT-related proteins. (**A**) Transwell assays were performed to analyse the effect of different treatments on the migration and invasion of Cal-27 and SCC-25 cells. (**B**,**C**) Changes in the expression level of cell migration-related proteins and the activity of the AKT signalling pathway after RCN1 knockdown detected by immunoblotting assay. *p < 0.05, **p < 0.01, ***p < 0.001.

To investigate the impact of RCN1 on OSCC cell migration and invasion, transwell assays were conducted. The findings demonstrated a significant decrease in the migration and invasion abilities of OSCC cell lines following RCN1 knockdown (Fig. 3A). Previous studies^22^ have revealed that epithelial-mesenchymal transition (EMT) contributes to tumour cell migration and invasion. Interestingly, after RCN1 knockdown, the expression of E-cadherin was found to be significantly increased, while the expression of N-cadherin and vimentin was significantly decreased (Fig. 3B,C). These results suggest that RCN1 knockdown may inhibit OSCC cell migration and invasion by modulating EMT-related proteins.

The AKT signalling pathway is known to play a crucial role in regulating various biological behaviours, including proliferation, migration, invasion, and apoptosis, in tumour cells^23–25^. It has also been extensively studied in the context of OSCC^26,27^. To further investigate whether the AKT pathway is involved in regulating the biological behaviours of OSCC cells by RCN1, the expression levels of AKT and phosphorylated AKT molecules were examined. The results revealed that total AKT expression remained unchanged after RCN1 knockdown. However, the phosphorylation level of AKT was significantly reduced. It is important to note that phosphorylated AKT represents activated AKT, indicating that the activation of AKT was inhibited following RCN1 knockdown (Fig. 3B,C). These findings suggest that RCN1 may be involved in the regulation of OSCC EMT through the AKT pathway.

### RCN1 contributes to M2-like polarization of macrophages

**Figure 4:**
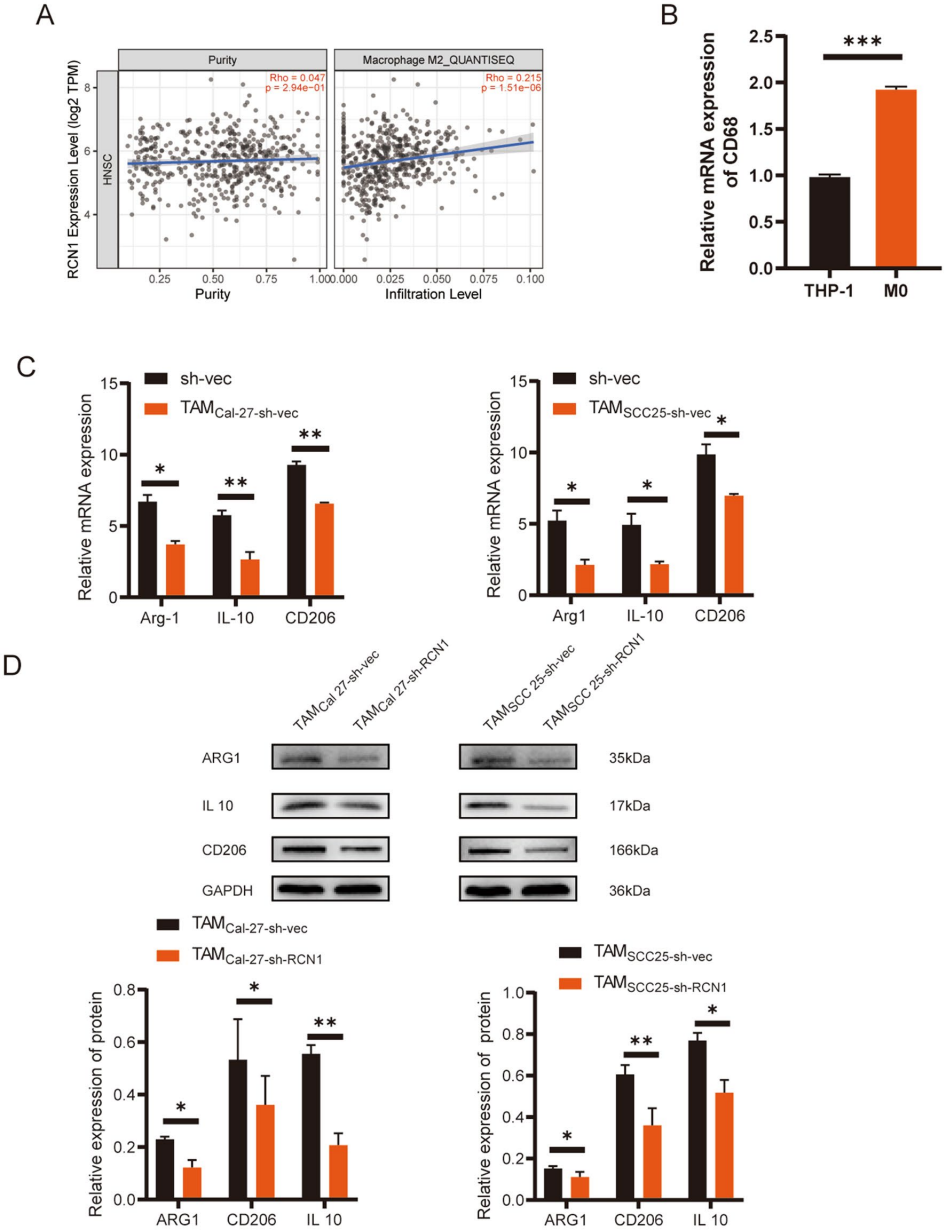
Knockdown of RCN1 in Cal-27 and SCC-25 cells inhibits M2 polarization of THP-1 macrophages. (**A**) The TIMER database was used to analyse the correlation between M2 macrophage infiltration and RCN1 expression in OSCC. (**B**) RT‒qPCR was used to detect the expression level of the M0 macrophage marker CD68. (**C**) RT‒qPCR was used to detect the expression level of the M2 macrophage marker expression in cocultured TAMs. (**D**) Protein levels of CD206, IL-10, and ARG1 in cocultured TAMs. *p < 0.05, **p < 0.01, ***p < 0.001.

In the tumour microenvironment, tumour cells have the capacity to recruit and influence macrophages, leading to their differentiation into M2 TAMs, which are known to have pro-tumorigenic effects in various cancers^28^. Analysis of the TIMER database revealed a significant positive correlation between RCN1 expression and the presence of M2 TAMs in OSCC (Fig. 4A). This suggests that RCN1 may be involved in promoting the formation of M2 TAMs. To further investigate this hypothesis, an in vitro coculture system was employed. THP-1 cells were stimulated with PMA to induce differentiation into M0 macrophages, as indicated by a significant increase in the expression of the marker CD68 (Fig. 4B). This confirmed the successful generation of M0 macrophages under PMA stimulation. To assess the impact of RCN1 on the ability of OSCC cells to induce macrophage polarization, supernatants from OSCC cell lines treated differently were cocultured with M0 macrophages. The results demonstrated that RCN1 knockdown significantly decreased the mRNA levels of CD206, Arg1, and IL-10 in M2 TAMs compared to the vector group (Fig. 4C). Furthermore, at the protein level, CD206, IL-10, and ARG1 expression was significantly decreased (Fig. 4D). These findings suggest that RCN1 knockdown prevents OSCC cells from inducing M2 polarization in TAMs. Thus, RCN1 appears to be a key regulatory molecule involved in OSCC cell-induced macrophage polarization.

## Discussion

OSCC has a high mortality rate due to high metastasis and recurrence rates and has become a major human health burden due to the lack of satisfactory treatment strategies^29^. Understanding the process of OSCC development and searching for key factors mediating its recurrence and metastasis have become important prerequisites for the development of effective targeted agents. In this study, we confirmed the inhibitory effect of RCN1 knockdown on OSCC progression and demonstrated, for the first time, that RCN1 can act as a key regulator of macrophage polarization in OSCC. This study therefore explores a novel mechanism by which RCN1 promotes tumour progression and suggests that RCN1 inhibition will be an effective strategy for the treatment of OSCC driven by high RCN1 expression.

RCN1 is an important biomarker for OSCC. Previously, some scholars^30^ found that RCN1 was highly expressed in the saliva of OSCC patients by collecting clinical saliva samples. We observed the aberrant expression of RCN1 in OSCC by bioinformatics analysis, and we found that its high expression was significantly associated with a poor prognosis in patients. We confirmed this finding by immunohistochemical staining. These results suggest that RCN1 may play a pro-cancer role in OSCC. This is consistent with previous findings that high expression of RCN1 is present in a variety of malignancies^8,10,31^.

RCN1 acts as a calcium-binding protein involved in the regulation of calcium ion flow. Calcium is a versatile intracellular signal. Studies have shown that when Ca^2+^ accumulates excessively in mitochondria, it leads to the development of apoptosis^32,33^. In our experiments, we also found that RCN1 knockdown inhibited the expression of Bcl-2 and caspase 3 and promoted cleaved caspase 3 expression. We inferred that RCN1 could regulate the apoptosis of OSCC cells. The growth of malignant tumours is facilitated by the rapid proliferation of tumour cells, and in our study, we found that RCN1 could promote tumour cell proliferation, which makes RCN1 an important molecule in regulating the development of oral cancer.

The AKT signalling pathway is widely activated in a variety of human cancers^34^. Snail is considered to be a potent repressor of E-cadherin and a downstream target of AKT. OSCC is a tumour of epithelial origin, and EMT is an important biological process for acquiring migratory and invasive capacity^35^. It has been shown that NETO2 activation of AKT pathway activity induces EMT, leading to oesophageal squamous cell carcinoma metastasis^36^. In hepatocellular carcinoma, RCN1 was found to promote metastasis of hepatocellular carcinoma cells by promoting EMT^8^. Our results demonstrated that RCN1 knockdown inhibited AKT activation, which stabilized snail and ultimately inhibited EMT. In addition, matrix metalloproteinase 9 (MMP9) is associated with malignant tumour progression and invasion. The results showed that RCN1 knockdown inhibited MMP9 expression. These results suggest that RCN1 knockdown may inhibit the migration and invasion of OSCC cells by inhibiting the phosphorylation of AKT pathway components.

TAMs are highly plastic^37^ and can differentiate into M1 and M2 types depending on the tumour microenvironment signals they receive. M1 TAMs play an antitumour role in cancer, whereas M2 TAMs, which are the main macrophages in the tumour microenvironment, have a role in blocking tumour immunity^38^. In glioblastoma, PD-L1 mediates immunosuppression by inducing M2 polarization of macrophages^39^. We found that M2 TAMs infiltration was positively correlated with RCN1 expression through an online database. Subsequently, we cocultured M0 macrophages with conditioned medium from the vector and knockdown groups and found that knockdown of RCN1 markedly suppressed its effects in promoting TAMs M2 polarization, suggesting that the expression of RCN1 in OSCC may alter the cellular properties that drive M2 polarization, but the mechanism needs to be further investigated.

In summary, our study demonstrated that high expression of RCN1 is associated with a poor prognosis in OSCC patients. We found that RCN1 induces the migration and invasion of OSCC cells through the AKT pathway. Additionally, we observed that low expression of RCN1 in OSCC cells inhibits M2 polarization of macrophages in the TME. Based on these findings, we conclude that RCN1 plays a significant role in promoting OSCC progression and holds promise as a therapeutic target. Our future research will focus on conducting animal experiments to further investigate the effects of RCN1 on tumours in vivo and explore the potential molecular mechanisms underlying RCN1-mediated TAMs M2 polarization in the TME. These experiments will provide deeper insights into the impact of RCN1 and offer a more comprehensive understanding of its role in OSCC progression.

## Conclusion

**Figure 5:**
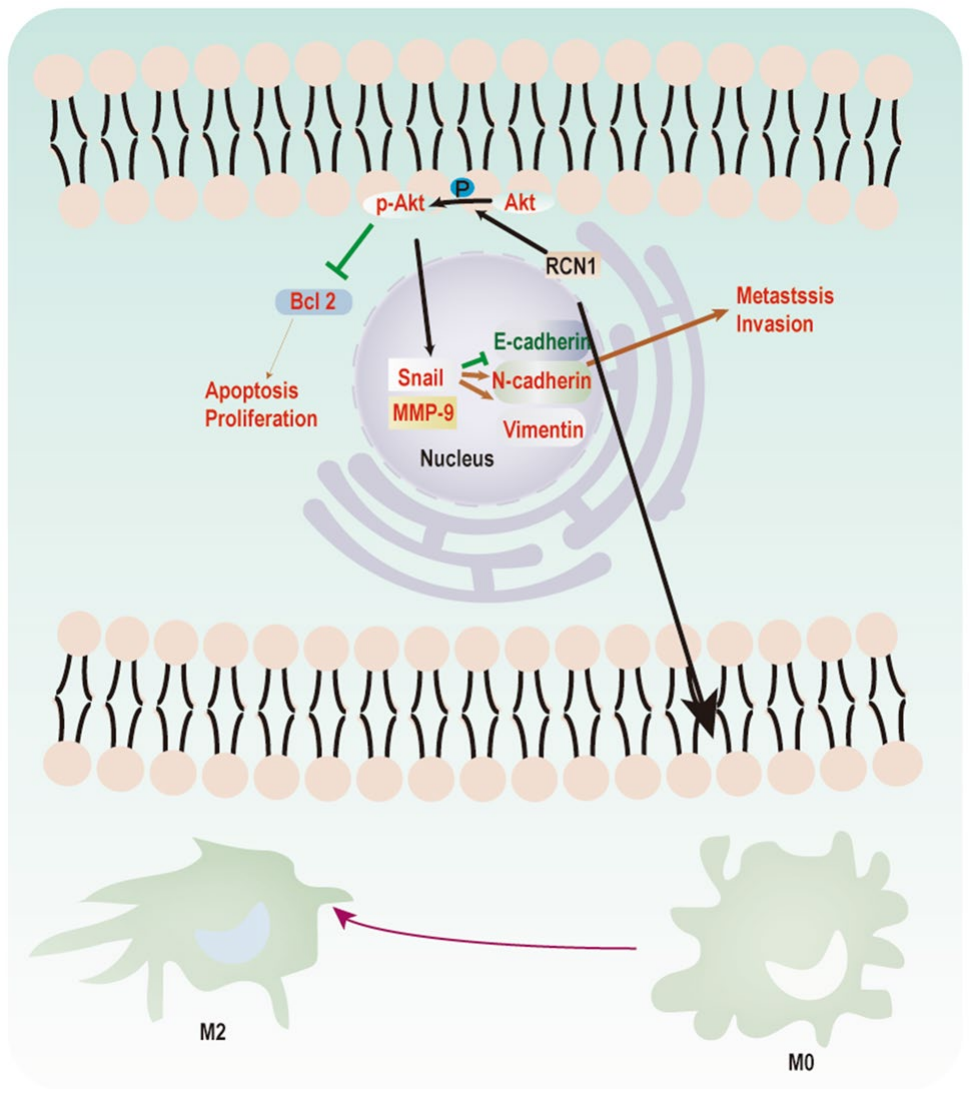
Mechanism diagram. RCN1 knockdown significantly inhibited the proliferation, migration, and invasion of Cal-27 and SCC-25 cells. Moreover, knockdown of RCN1 in Cal-27 and SCC-25 cells inhibited the M2 polarization of THP-1 macrophages.

As depicted in Fig. 5, RCN1 deficiency not only inhibited proliferation, migration, and invasion but also induced apoptosis in Cal-27 and SCC-25 cells. Additionally, knockdown of RCN1 in Cal-27 and SCC-25 cells resulted in inhibition of the M2 polarization of THP-1 macrophages.

### Supplementary Information

Supplementary Information.

## Data Availability

All raw data are publicly available from corresponding databases. Processed data are available upon reasonable request from the corresponding author.
