# Supplementary material for: RCN1 deficiency inhibits oral squamous cell carcinoma progression and THP-1 macrophage M2 polarization
Source: Sci Rep. 2023 Dec 6;13:21488. doi: 10.1038/s41598-023-48801-2 (PMC10700561; doi:10.1038/s41598-023-48801-2)
Supplement: Supplementary file 1 — Supplementary Information. [file 41598_2023_48801_MOESM1_ESM.pdf]

RCN1 deficiency inhibits oral squamous cell carcinoma progression and THP-1 M2 macrophage polarization

Han Liu[1,2#], Haiyang Guo[3#], Yuehan Wu[1,2], Qiannan Hu[1], Guangbing Hu[4], Huan He[1], Yaolin Yin[4], Xiaoxu Nan[1,2], Gaoren Lin[1,2], Jinpeng Han[1,2], Runzhe Zhao[1,2], Ying Liu[1,2*]

[1] Department of Stomatology, Affiliated Hospital of North Sichuan Medical College, Nanchong, China.

[2] Department of Stomatology, North Sichuan Medical College, Nanchong, China.

[3] Digestive Endoscopy Center, Affiliated Hospital of North Sichuan Medical College, Nanchong, Sichuan, China

[4] Institute of Hepato-Biliary-Pancreatic-Intestinal disease, North Sichuan Medical College, Nanchong, China

[#]Contributed equally

* Corresponding author. Ying Liu, Department of Stomatology, Affiliated Hospital of North Sichuan Medical College, Nanchong, China.

E-mail address: liuying08_nsmz@163.com

Supplementary chart

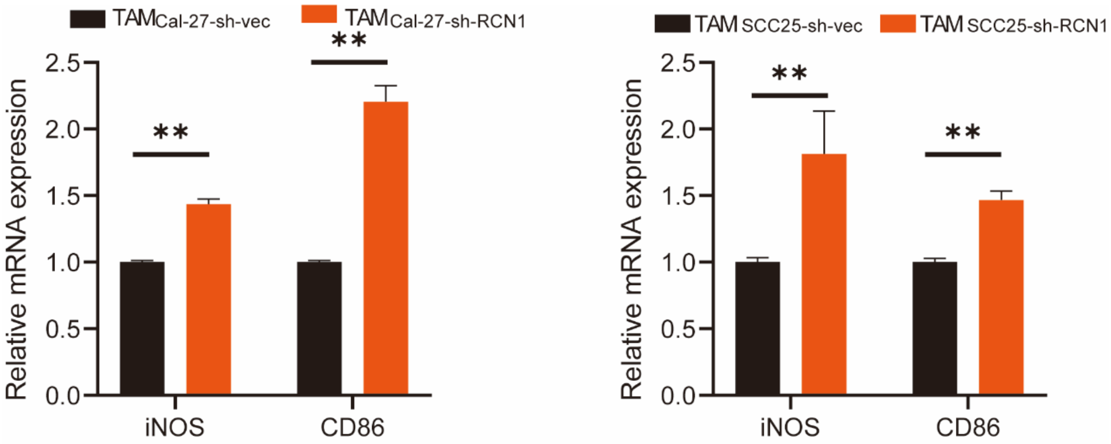

Knockdown of RCN1 in Cal-27 and SCC-25 cells promotes M1 polarization of THP-1 macrophages. (A) RT–qPCR was used to detect the expression level of the M1 macrophage marker expression in cocultured TAMs. *$p < 0.05$, **$p < 0.01$, ***$p < 0.001$.
